# Towards Critical Analysis of the Political Determinants of Health: Comment on "How Neoliberalism Is Shaping the Supply of Unhealthy Commodities and What This Means for NCD Prevention"

**DOI:** 10.15171/ijhpm.2019.102

**Published:** 2019-11-02

**Authors:** Julia Smith

**Affiliations:** Simon Fraser University, Burnaby, BC, Canada.

**Keywords:** Neoliberalism, Governance, Politics, Global Health, Critical

## Abstract

The recent perspective article "How Neoliberalism Is Shaping the Supply of Unhealthy Commodities and What This Means for NCD Prevention," by Lencucha and Throw, interrogates how the dominant neoliberal paradigm restricts meaningful policy action to prevent non-communicable diseases (NCDs). It contributes an NCD perspective to the existing literature on neoliberalism and health, which to date has been dominated by a focus on HIV, gender and trade agreements. It further advances the emerging commercial determinants of health (CDoH) scholarship by calling for more nuanced analysis of how the governance of both health and the economy facilitates corporate influence in policy-making. In political science terms, Lencucha and Throw are calling for greater structural analysis. However, their focus on the pragmatic, as opposed to political, aspects of neoliberalism reflects a hesitancy within health scholarship to engage in political analysis. This depoliticization of health serves neoliberal interests by delegitimizing critical questions about who sustains and benefits from current institutional norms. Lencucha and Throw’s call for greater interrogation of the structures of neoliberalism forms a basis from which to advance analysis of the political determinants of health.

## Introduction

In 1848, Rudolf Virchow, a cellular pathologist and public health pioneer, wrote, “Medicine is a social science, and politics nothing but medicine at a larger scale.”^1^ Despite being one of most frequently quoted one-liners in public health, the relationship between politics and health remains neglected in health scholarship. The perspective article “How Neoliberalism Is Shaping the Supply of Unhealthy Commodities and What This Means for NCD Prevention” by Lencucha and Throw takes a step to rectifying this gap by interrogating how the dominant neoliberal paradigm restricts policy action to prevent non-communicable diseases (NCDs).^2^ It contributes to literature on neoliberalism and health and the commercial determinants of health (CDoH). However, further critical analysis is needed to advance understanding of political determinants of health, which are the “norms, policies and practices that arise from transnational interaction … that cause and maintain health inequities.”^3^

The commentary contributes an NCD perspective to the existing literature on neoliberalism and health, which to date has been dominated by a focus on HIV, gender and trade agreements. The HIV epidemic, in particular, catalyzed critical analysis of how neoliberal policies exacerbated health inequities. Take Msimang’s description of the links between mining development and the spread of the HIV epidemic in South Africa:

“If there was a recipe for creating an AIDS epidemic in Southern Africa, it would read as follows: ‘Steal some land and subjugate its people. Take some men from rural areas and put them in hostels far away from home, in different countries if need be. Build excellent roads. Ensure that the communities surrounding the men are impoverished so that a ring of sex workers develops around each mining town. Add HIV.”^4^

Also writing from a feminist perspective, but focused on the response as opposed to the drivers to HIV, O’Manique argues neoliberal assumptions prioritize interventions focused on individual behavior change and fail to recognize the unpaid care work of women.^5^ Scholarship on the HIV response has also advanced analysis of how free trade agreements, informed by neoliberal ideals, influenced who has access to life prolonging medications and who does not.^6^ More recent research documents how corporations use trade agreements to oppose public health policies, such as the standardized packaging of tobacco products, and to facilitate market expansion for unhealthy products.^7^ While political scientists and ethicists have contributed further theoretical insights on neoliberalism influences over health and within health governance,^3^ the public health literature remains focuses on case studies or select actors.

There has been an increasing in interest in the CDoH - the activities of profit seeking entities that effect health outcomes.^8^ Kickbush et al’s framework identifies drivers (such as internationalization of trade and capital), channels (such as marketing and lobbying) and outcomes (related to policy environment, consumers and health) of CDoH.^9^ Lencucha and Throw’s analysis particularly advances discussion on, and stresses the importance of, the global drivers. It illustrates how neoliberal norms and institutions influence perceptions of the ‘proper’ relationship between government and market (small government and free markets); the perceived need to ‘balance’ economic and health interests (with the scales weighted in favour of economic gain); and the ‘right’ of corporations to participate in decision-making around health (such as product regulation). Notably, it calls for greater research into the supply-side of product environments, an aspect neglected in the CDoH literature, which is predominantly focused on the marketing and lobbying activities of corporations. This is an important call for nuanced analysis of how the governance of both health and the economy facilitates corporate influence in policy-making.

In political science terms, Lencucha and Throw are arguing for a focus on structure, as well as agency. Structure refers to the macro forces, such as culture and political ideologies, that construct the parameters of choice and action, while agency refers to actors’ capacity to act upon a situation as relatively autonomous individuals.^10^ Social scientists have spent much energy debating whether structure or agency is the dominant force in social and political life. Nuanced analyses note that structure and agency are two sides of the same coin, advocating for greater interrogation of the relationship between them.^11^ Lencucha and Throw point out that the majority of literature on NCD prevention and policies has focused on actors, neglecting analysis of the structures they operate within. They take a step towards rectifying this imbalance by identifying neoliberalism as the dominant structural force that limits options within NCD health policy-making.

However, their “focus on the paradigmatic (rather than political) aspect of neoliberalism” limits analysis of the power relationships that create, sustain and benefit from these structures.^2^ Indeed, it is arguable whether the pragmatic and political aspects of neoliberalism can be separated.^12^ Neoliberalism is “a political project” developed from ideologies aimed at reconfiguring state roles, increasing the power of private actors and limiting acceptable forms of global governance.^13^ As Gill and Benatar write, “neoliberal capitalism is not just a set of economic processes but also a system of power. This system does not involve the accumulation of goods to improve livelihood and social wellbeing, but is driven by the accumulation of monetary values (exchange values) for profit.”^14^ Ignoring this agenda, and how it contradicts health equity goals, results in an analysis that, while clearly describing neoliberalism’s rise and functions, does so as if neoliberal institutions just appeared, sustained by some mysterious force. It is like describing the workings of a machine, without mentioning its purpose or the type of fuel it runs on.

This approach reflects a hesitancy in global health scholarship to engage in critical political analysis. Kay and Williams write that literature on global health governance in general “has largely failed to ground analysis of global health issues and outcomes within the broader political economic project of globalization.”^15^ While, as outlined above, there is an increasing body of research on neoliberalism and health, much of this comes from feminist and political economy scholars. Analysis of the intersections between politics and health remains peripheral in global and public health fields. For example, the first global health conference focused on political economy, the Prince Mahidol Award Conference in Thailand, was held in 2019.^16^ The Lancet-University of Oslo Commission Report on Global Governance for Health, despite the stated focus on governance (a political process), failed to engage with questions about power or make recommendations that would challenge the ways dominant neoliberalism restricts health governance options.^17^

Yamin et al write, health has been “depoliticized by the disciplinary orthodoxies of medicine and public health.”^18^ The dominant culture of positivistic scientific inquiry, drawn from the medical field, guides the study of health policy and governance.^19^ Within this orthodoxy ill health is not only predominately defined as an individual biological problem with a technical solution, but public health scholarship is dominated by empirical analysis of observable patterns, cause and effect.^20^ For example, Gill and Benatar argue the Consortium of Universities for Global Health is “steeped in the biomedical model of disease.”^3^ While suitable for medical research, this model cannot interrogate the political ideologies and power structures that shape health policy-making, or identify how they have evolved from legacies of imperialism and colonialism.^17^ This contradiction – between the positivistic ideals of public health research and the need for critical analysis of health policy and governance – discourages research on the political determinants of health.

Which in turn serves neoliberal interests. Kay and Williams write of an “enduring ideational alliance of neoliberalism and the biomedical model,” which melds medical discourse with the interests of global capital.^15^ This alliance not only defines ill health as primarily an issue of resources requiring technical and market-based solutions, it delegitimatizes analysis that asks critical questions about health policy and governance.^21^ For, as Bambra et al write, the depoliticization of health “does not occur by chance: both the masking of the political nature of health, and the forms of the social structures and processes that create, maintain and undermine health, are determined by the individuals and groups that wield the greatest political power.”^19^ In other words, the depoliticization of health serves powerful interests by delegitimizing analysis that might reveal and question those interests.

Despite shying away from political analysis, Lencucha and Throw’s Perspective does call for critical interrogation of the structures of neoliberalism. They advocate “systematic engagement with the assumptions that continue to structure institutions across sectors.”^2^ The process of identifying and interrogating assumptions facilitates awareness of the constraints actors operate within, which in turn enables opportunities to challenge these, expose contradictions (for example, by raising questions about why commercial actors often have input into health policies, but health actors rarely have input into commercial policies), and pose alternatives (such as allowing health actors equal input into commercial policies). The next step is then to generate alternatives. For example, Benatar et al propose what they term “development of sustainability” as an ethical framework and political ideology based on human rights, social democracy and social justice.^22^

Mackenbach rephrases the quote from Virchow noted at the start as, “human health and disease are the embodiment of the successes and failures of society as a whole, and the only way to improve health and reduce disease is by changing society by, therefore, political action.”^1^ If, as Lencucha and Throw convincingly illustrate, neoliberalism is THE structural barrier to NCDs prevention, then health scholars need to not only describe the limits it imposes, but also challenge them through critical analysis of the political determinants of health.

## Ethical issues

Not applicable.

## Competing interests

Author declares that she has no competing interests.

## Author’s contribution

JS is the single author of the paper.
